# Potato Disease

**Published:** 1846-06

**Authors:** 


*Potato Disease.*—Dr. Playfair, in a lecture lately delivered to the Royal Agricultural Society, at the Royal Institution, stated, that he concluded from all his researches into the potato disease, that the potato labored under *dropsy!*—a singular enough conclusion for a chemist to draw; for a sound and a diseased potato, each dug from the same locality or root, when sliced and dried, will be found to contain as nearly as possible a like proportion of moisture.—*Ibid.*

